# Designing a multi-epitope construct using immuno-informatic tools to prepare a messenger RNA vaccine against *Rhipicephalus microplus* ticks

**DOI:** 10.14202/vetworld.2024.2235-2247

**Published:** 2024-10-07

**Authors:** Ubaid Ullah, Kamran Ashraf, Wasim Shehzad, Muhammad Imran Rashid

**Affiliations:** 1 Department of Parasitology, University of Veterinary and Animal Sciences, Lahore 54200, Pakistan; 2 Institute of Biochemistry and Biotechnology, University of Veterinary and Animal Sciences, Lahore 54200, Pakistan

**Keywords:** immuno-informatic tools, lipid nanoparticles, multiepitope construct, *Rhipicephalus microplus*

## Abstract

**Background and Aims:**

Ticks are blood-feeding ectoparasites that transmit pathogens to animals and humans. One of the most important hard ticks in animals is *Rhipicephalus microplus*, which transmits *Babesia* and *Anaplasma* spp. Although many potential tick vaccine candidates have been identified, no effective vaccine that can provide sterile immunity against *R. microplus* tick infestations has been developed. This study aimed to design a construct using different computational tools to identify and predict immunogenic epitopes within protein sequences and to prepare a messenger RNA (mRNA) vaccine against *R. microplus* based on lipid nanoparticles (LNPs).

**Materials and Methods:**

The *R. microplus* proteins (Bm86, Subolesin, and ATAQ) were selected and their consensus sequence was obtained from the National Center for Biotechnology Information in FASTA format. The Immune Epitope Database and Analysis Resource (IEBD) server was used for the prediction of helper T-cell epitopes, the NetCTL 1.2 server was used to predict cytotoxic T-cell epitopes, and the ABCpred server was used for B-cell epitope prediction. Antigenicity testing, allergenicity assessment, and toxicity screening were immuno-informatic techniques used to identify potent epitopes within protein sequences. The multi-epitope construct was prepared and cloned into the pVAX1 plasmid. Plasmids were transformed in compatible competent cells, and restriction analysis was performed. After restriction analysis of the transformed plasmid, *in vitro* transcription was performed to prepare mRNA. The mRNA was purified, quantified, and converted into complementary DNA, and gene-specific primers were used to confirm *the in vitro* transcription of mRNA. A mixture of four lipids containing 1,2-dioleoyl-3-dimethylammonium-propane (DODAP), Distearoylphosphatidylcholine (DSPC, cholesterol, and 1,2-Dimyristoyl-sn-glycero-3-methoxypolyethylene glycol-2000 (DMG PEG-2000 was used to prepare LNPs. LNPs were characterized using a scanning electron microscope, Zeta potential, and Zeta Sizer tests.

**Results:**

More than 1000 epitopes were predicted, from which only nine helper T-lymphocytes, 18 cytotoxic T-lymphocytes, and nine B-cell epitopes of all three proteins were selected with high antigenic scores of 0.958 for Bm86, 0.752 for Subolesin, and 0.964 for ATAQ, respectively. An adjuvant was used to enhance immune responses, all of which were linked to one another using GPGPG, AAY, and KK linkers, respectively. The physiochemical properties predicted that the instability index of the construct would be <40%, indicating that the construct is stable. Plasmids were transformed in compatible competent cells, and white-transformed colonies were observed. Restriction analysis was performed, DNA was transcribed into mRNA, and LNPs were prepared and characterized.

**Conclusion:**

More than 1000 epitopes were predicted using immune informatic tools, and only high-scoring epitopes were selected. A multi-epitope construct was designed using bio-informatic tools, and its physicochemical properties were predicted. The design construct was inserted into the pVAX1 plasmid, and *in vitro* transcription was performed to prepare the mRNA. LNPs of mRNA were prepared and characterized to be used as vaccines. It was found that LNPs were stable and nanometer-sized.

## Introduction

Ticks are blood-feeding ectoparasites that transmit pathogens to animals and humans worldwide [1]. It acts as a vector for transmitting viruses, bacteria, and parasites in animals, which mainly affects the livestock sector globally [2]. Ticks directly impact animals’ health by causing blood loss, reducing animal meat and milk production, and indirectly by transmitting pathogens that cause various tick-borne diseases (TBDs) in animals, such as Babesiosis, Theileriosis, and Anaplasmosis [3]. Production losses due to TBDs are billions of dollars globally, and most these losses occur in developing countries [4]. Several studies have shown that >80% of cattle are infected by ticks and TBDs [5]. Throughout the world, 100,000 cases of illness are reported annually from transmitted pathogens from ticks. Ticks are the second largest mosquito vectors of human and animal diseases [6]. The most common hard tick is *Rhipicephalus microplus*. There are different methods for controlling ticks and TBDs. In the traditional method, chemical acaricides are the main components [7]. The irrational use of chemical acaricides against ticks in animals leads to resistance, environmental contamination, and decreased milk and meat quality [8]. Therefore, the irrational use of chemical acaricides should prevent the reduction of tick resistance to such agents. The development of new acaricides is an expensive and time-consuming process. Alternative approaches to tick control include immunological techniques such as vaccines and biological control [9]. The vaccine is the best immunological control system for ticks and TBDs [10]. It is cost-effective and environmentally friendly. It is an alternative to chemical acaricides that are not environmentally friendly. There is no risk of tick resistance to vaccine like in chemical acaricides [9]. In 1990, a recombinant tick vaccine targeting *R. microplus’s* Bm86 gut-antigen was developed and commercialized [11–14]. These vaccines have the potency to protect against and trigger an immune response [13]. They were registered and commercialized in Australia (TickGARD^®^, Commonwealth Scientific and Industrial Research Organisation (CSIRO) in collaboration with Biotech Australia and Latin American countries (Gavac^®^, Heber Biotech, Havana, Cuba) from 1993 to 1997, but these have low efficacy [15].

The multiple epitope vaccine is another type of vaccine that effectively elicits an immune response in the host. The B-cell, t-helper, and cytotoxic t-cell epitopes were combined through linkers, and a multi-epitope vaccine was designed to treat Crimean-Congo hemorrhagic fever [16]. A previous study by Suleman *et al*. [17] showed that potential vaccine candidate antigens were selected, and a multi-epitope vaccine was designed. In another study, a multi-epitope construct was designed by combining different epitopes of proteins (helper T-lymphocyte [HTL], cytotoxic T-lymphocyte [CTL], and B-cell) [18]. The epitopes of Bm86 and Subolesin proteins were predicted and used to prepare a multi-epitope vaccine that effectively eradicated *R. microplus* [19]. Recombinant vaccines also reduce tick infestation and resistance but are lengthy and expensive procedures. Another approach to overcoming this issue is the messenger RNA (mRNA) vaccine. The mRNA vaccine is more effective and has fewer chances for mutation because it is not a DNA-based vaccine [20]. The mRNA vaccine against ticks was developed, and its efficacy was evaluated in guinea pigs and rabbits by giving tick challenges and using enzyme-linked immunosorbent assay [21]. mRNA is fragile and can be degraded easily by extracellular enzymes when inoculated into animals. Therefore, it is necessary to prepare lipid nanoparticles (LNPs) of mRNA before inoculation into experimental animals. LNPs were prepared using four different lipids: cationic, phospholipid, PEGylated, and cholesterol. A lipid stock solution containing ionizable lipids, DSPC, cholesterol, and PEG was prepared [22]. A similar study was conducted to prepare LNPs using different lipids and mRNAs, and the nanoparticles were then characterized [23].

In this study, the *R. microplus* targeted proteins (Bm86, Subolesin, and ATAQ), HTL, CTL, and B-cell epitopes were identified and characterized using a range of immune epitope database tools, and a multi-epitope construct was designed. The designed vaccine construct was evaluated in nature and was found to be both antigenic and non-allergic. The physicochemical properties of the proposed construct were evaluated using the ProtParam server. The construct was predicted to be stable and antigenic, non-allergenic, thermostable, and hydrophilic, which are the defining characteristics of the designed construct. The construct is hydrophilic, as demonstrated by its GRAVY value; thus, it interacts with an environment that is predominantly composed of water [24]. *In silico* cloning, a compatible plasmid was used, and the DNA was transcribed into mRNA. Because mRNA is fragile, LNPs of transcribed mRNA were prepared using a lipid mix and characterized using a scanning electron microscope (SEM), Zeta sizer, and Zeta potential tests. These findings suggest that the multi-epitope construct designed in this study can be used as a vaccine candidate against *R. microplus* ticks, enabling a more effective control of infestations. For the first time, we have shown that tick vaccines can be designed by selecting the immunogenic epitopes of different proteins using immuno-informatics tools.

This study aimed to prepare a multi-epithelial vaccine construct using bioinformatic tools and transcript it into mRNA through *in vitro* transcription. This mRNA can be used to vaccine *R. microplus* ticks.

## Materials and Methods

### Ethical approval

The study was not based on living animals so, ethical approval was not necessary.

### Study period and location

The study was conducted from June-2022 to June-2023 in the Department of Parasitology, University of Veterinary and Animal Sciences, Lahore, Pakistan.

### Selection of *R. microplus* proteins

The *R. microplus* proteins (Bm86, Subolesin, and ATAQ) were selected and their consensus sequences were used to construct multi-epitope antigens. These three proteins were selected based on previous reports and their immunogenicity levels [19]. The previously reported sequences of these proteins were obtained from the National Center for Biotechnology Information (NCBI) using FASTA format (bioinformatic tool for consensus sequence). The scratch protein predictor server was used to predict antigenicity, epitope prediction, tertiary protein structure, secondary structure, solubility, and protein overexpression (https://scratch.proteomics.ics.uci.edu/) [25].

### Prediction of epitopes

Different computational tools were used to identify and predict immunogenic epitopes within protein sequences. B- and T-cell epitopes were predicted using different servers.

### Prediction of T-helper, cytotoxic T-cell, and B-cell epitopes

The Helper T-cell epitopes of the three target proteins were identified using the Immune Epitope and Database Analysis Resource (IEDB) server (http://tools.iedb.org/mhcii/). The human leukocyte antigen (HLA) allele of major histocompatibility complexes (MHC) II was used as a reference allele because it accounts for 99.9% of all allele distributions. This server predicts HTL epitopes based on low half-maximal inhibitory concentration values and percentile rank while leaving all other variables at their default levels. Epitopes with strong binding abilities to MCH II receptors have been identified [26]. The CTL epitopes recognized by MHC class I supertypes A2, A3, and B7 were predicted using the NetCTL 1.2 server (https://services.healthtech.dtu.dk/services/NetCTL-1.2/) [27]. The predicted CTL epitopes were further perused for immunogenicity evaluation using IEDB class I immunogenicity [28]. The epitopes with higher scores were considered to have significant binding affinity and were thus selected for the design of vaccines. The ABCpred server was used to predict B-cell epitopes [29]. The ABCpred server () helps identify epitopes in protein regions that can be used to select candidates for multi-epitope vaccines.

### Epitope selection

Every epitope of each protein (Bm86, Subolesin, and ATAQ) was checked through Allertop, Thinprep, and VaxiJen 2.0 servers (https://www.ddg-pharmfac.net/vaxijen/VaxiJen/VaxiJen.html) to determine the allergic, toxic, and antigenic potential of each epitope. Non-allergic epitopes, non-toxic, and had high antigenicity scores were selected. The ToxinPred 2.0 server (https://webs.iiitd.edu.in/raghava/toxinpred/multi_submit.php) was used to predict epitope toxicity levels. Non-toxic epitopes were selected and used for multiepitope vaccine design [30]. The VaxiJen 2.0 server was used to predict epitope antigenicity. High-score level epitopes (above 0.5 scores) were selected and used for the multi-epitope vaccine design. The Allertop v. 2.0 server (https://www.ddg-pharmfac.net/AllerTOP/) was used to predict epitope allergic levels [30]. Non-allergic epitopes were selected and used for multi-epitope vaccine design.

### Construction of a multi-epitope vaccine construct

Finally, a multi-epitope-based construct was designed using previously reported approaches, including selected HTLs, CTLs, and B-cell epitopes [31]. Suitable linkers were added to provide amino acid flexibility; these linkers were required to fold into desirable conformations. The adjuvant 50S ribosomal protein L7/L12 was added to enhance immunity and increase the effectiveness of the vaccine during immunization. This adjuvant can act as an agonist for toll-like receptors and could induce antigen-specific immune responses on immunization [32, 33]. We used the 50S ribosomal L7/L12 UniProt database (P9WHE3) with a 130-amino-acid long, which was connected to the N-terminus, as an adjuvant using the linker glutamic acid, alanine, alanine, alanine, lysine. Adjuvants improve efficacy by increasing immunogenicity. Gly-Pro-Gly-Pro-Gly linkers are employed to connect HTL epitopes, lysine-klysine linkers to B cells, and alanine-alanine-tyrosine linkers to connect CTL epitopes [34, 35].

### Predicting physicochemical properties, immunogenicity, and allergenicity

The antigenicity and immunogenicity of the vaccine constructs were determined using ANTIGENPro [25] and VaxiJen 2.0 server [36], respectively. The model predicts antigenicity based on autonomous alignment. Immunogenicity refers to a vaccine’s ability to prime the immune system to respond strongly to both outside invaders and additional vaccines. The ExPASY Protparam server (https://web.expasy.org/protparam/) was used to evaluate the physicochemical features of the multi-epitope-based prepared construct [37].

### *In silico* cloning

The final designed construct, which was evaluated using different immuno-informatic tools, was inserted into the plasmid pVAX1 (Cat. No: U9235IA050, GeneScript^®^, USA) between the *BspQ1* and *Nde1* restriction sites. The plasmid (SC1691 Express Cloning: Rm_pVAX1- *BsaI-SpeI*-*SapI-BspMI*-freeTerminator-T7 deleted-100A-BspQI Plasmid) was designed and ordered from GeneScript^®^. Poly (A) (SC2332: Poly (A) Guarantee Poly (A) detected, 91~110, A = N ± 5nt) was added to the plasmid to stabilize the gene of interest. The 5′ and 3′ untranslated regions (UTR) were also added to the plasmid to prevent mRNA degradation after *in vitro* transcription. The pVAX1 plasmid has a NeoR/KanR antibiotic-resistant region, the cytomegalovirus promotor region, and the gene of interest (2227 bp).

### Competent cell preparation and transformation

Calcium chloride-based Dh5α competent cells were prepared using an improved method of competent cell preparation [38]. Heat shock was used to transform plasmids in calcium chloride-competent cells [39]. The 1 μL of plasmid (100 ng) and competent cells were thawed on ice for 15 min. The plasmid was mixed in 50 μL Dh5α competent cells in the presence of flame to avoid contamination. Then, a vial containing plasmids mixed in competent cells was kept in a water bath at 42°C for 90 s and placed back on ice and 600 μL of fresh luria-bartani broth medium (Catalog No. CM0996, Oxoid Ltd, UK) was added to it as a nourishing medium. The positive vial in which plasmid was added and the negative vial in which no plasmid was added and was placed in a shaker incubator at 37°C and 220 rpm for 40 min. The 200 μL of the solution was poured on a kanamycin-resistant agar plate and kept in an incubator at 37°C overnight.

### Plasmid extraction and digestion

After transformation, the plasmid was extracted using a GeneJET Plasmid Miniprep kit (Cat. No: 2680476, Thermo Fisher Scientific, USA). The extracted plasmid was run on a 1% agarose gel at 120 V and 400 W for 30 min. A gel doc (Thermo Fisher Scientific, Ibright750) was used to visualize the plasmid. After plasmid extraction, restriction analysis of the plasmid was performed for single, double, and triple digestion. The enzymes *Xho1* and *BspQ1* were used for linearization of the plasmids, *Nde1* and *Xho1* were used for double digestion, and *Acc1* was used for triple digestion. *Acc1* enzymes cut plasmids from three different sites. Plasmids, enzymes, and their compatible buffer were incubated at 37°C for 40 min.

### *In vitro* transcription

The linearized plasmid was transcribed into mRNA according to kit T7ultra mMESSAGE mMACHINE (Cat. No: AM1345, Thermo Fisher Scientific). Enzyme RNA polymerase was used for the *in vitro* transcription of the plasmid into mRNA. All the reagents were thoroughly and gently mixed. The reaction mixture was incubated at 37°C for 2–3 h. After incubation, mRNA was treated with Turbo DNase (Cat. No: AM1345, Thermo Fisher Scientific) to avoid DNA contamination and proteinase-k (100–200 μg/mL) and 0.5% sodium dodecyl-sulfate for protein contamination. Then, the samples were kept at 50°C for 30 min. Phenol-chloroform isopropanol was then performed to elute RNA using the kit method MEGAclear (Cat. No: AM1908, Thermo Fisher Scientific).

### Purification and quality assessment of mRNA

The transcribed mRNA was purified using a MEGA clear kit MEGAclear (Cat. No: AM1908, Thermo Fisher Scientific). The purified mRNA was used for complementary DNA (cDNA) preparation using a kit (Cat. No: 18064-022, Thermo Fisher Scientific) to verify the *in vitro* transcription of the plasmid using gene-specific primers. The quality and quantity of mRNA were determined using nanodrops of mRNA.

### Preparation of cDNA and PCR

The cDNA was synthesized by superscript reverse transcriptase kit (Cat. No: 18064-022, Thermo Fisher Scientific) from the mRNA. The mRNA was converted into first strand DNA, and then using gene-specific primers, we prepared second strand DNA. The gene-specific primers were designed using Prim3way software. The forward primer has the sequence 5’-AAACCTTTGAAGTGACCGCG-3’ and the reverse primer has the sequence 5′-TTATACACGCATTCCTGGCC-3′, and the final expected product is 1855 bp.

### Preparation and characterization of LNPs

Four lipids were used as lipid mixtures. DODAP (Cat. No: HY-130751, MedChem Express, USA), DSPC (Cat. No: HY-W0401930, MedChem Express, USA), Cholesterol (Cat. No: C3045-25G, MedChem Express, USA), and DMG PEG-2000 (Cat. No: HY-112764, MedChem Express, USA). A 25 mM lipid stock solution containing 50:10:38.5:1.5% ionizable lipid: DSPC: Cholesterol: PEG was prepared [22]. Lipid mixture: mRNA, 1:5 was used for LNP preparation. LNPs were prepared by hand mixing [40]. The lipid mixture was brought to room temperature (25°C) before use and vortexed as needed to mix. The mRNA was thawed on ice. After thawing, mRNA was added to the desired concentration in sodium acetate buffer (0.4 μM). The lipid mixture was added to the mRNA solution and mixed rapidly. The solution became cloudy and opaque, and it was diluted (~20×) to PBS to neutralize the pH. After the LPNs were prepared, they were characterized using a SEM, Fourier transform infrared, Zeta sizer, and Zeta Potential.

The zeta sizer and zeta potential (Litesizer 500, Antoon Paar Graz, Austria) were obtained from the Entomology Laboratory, Department of Parasitology University of Veterinary and Animal Sciences, Lahore. The SEM (Nova SEM 450, Thermo Fisher Scientific) test was performed at the Lahore University of Management Sciences.

## Results

### Select proteins of *R. microplus*

The *R. microplus* protein Bm86, Subolesin, and ATAQ amino acid sequences were retrieved from the NCBI database and analyzed using the scratch protein predictor; a score of >0.5 was considered immunogenic. The precision of this approach exceeded 75%. All three proteins were antigenic, with antigenic scores >0.70 (Table-1).

**Table-1:** *Rhipicephalus microplus* proteins predicted antigenic score.

Proteins	Accession no.	Predicted antigenic score	Thinprep and Allertop
Bm86	QOE77966	0.958	Nontoxin and nonallergic
Subolesin	AFQ91885	0.752	Nontoxin and nonallergic
ATAQ	A0A223PJI9	0.964	Nontoxin and nonallergic

### HTL epitope identification

Prominent in triggering B-cell activation and cytotoxic T-cell responses to foreign antigens, HTL epitopes also play key roles in boosting adaptive immune responses. This study predicted more than 1000 (15-mer) HTL epitopes using the IEDB-recommended 2.22 prediction methods. The top nine most immunogenic epitopes were selected based on their antigenicity values and percentile ranks at 5%. These nine epitopes were further used to construct a multi-epitope-based vaccine construct (Table-2).

**Table-2:** Selected potential HTL epitopes of three *Rhipicephalus microplus* proteins.

Protein name	Selected allele	Predicted epitope	Percentile rank score	Score
Bm86	HLA-DRB1*01:01	IALFVAAVSLIVECT	0.67	1.034
		GIALFVAAVSLIVEC	0.56	0.845
		HPIGEWCMMYPKLLI	4.6	0.523
Subolesin	HLA-DRB1*01:01	AGNYTLRCALRSAGA	4.90	0.771
		GNYTLRCALRSAGAE	4.70	0.913
		IVIGILIPAVIVVIL	4.2	1.698
ATAQ	HLA-DRB1*01:01	NLYGKSLMATRLLKC	0.42	1.226
		GNYTLRCALRSAGAE	0.45	0.913
		QSAMKNLYGKSLMAT	0.02	0.714

### CTL epitope identification

Twenty-seven CTL epitopes were predicted from three *R. microplus* proteins using the NetCTL 1.2 server. We selected three MHC class-I Supertypes A2, A3, and B7 for epitope prediction because they comprise 88.3 % of the global population. Specifically, CTL epitopes with scores of 0.60 were used in this study. Only 27 epitopes were identified with a score of 0.60, and only 18 CTL epitopes were highly immunogenic. These variables were used in the vaccine design (Table-3).

**Table-3:** Selected potential CTL epitopes of *Rhipicephalus microplus*.

Protein	supertype A2		supertype A3		supertype B7	
		
	Predicted epitope	Scoring	Selected epitope	Scoring	Predicted epitope	Scoring
Bm86	LLNEYYYTV	1.461	TTAATTTTK	1.434	CPSGSTVAE	0.755
	FVAAVSLIV	1.244	LIAEKPLSK	1.414	AAVSATGLL	1.243
	YTVSFTPNI	1.154	CLRPDLTCK	1.038	AVSATGLLL	1.172
Subolesin	LLNEYYYTV	1.461	RMMKERESK	1.211	RSPKRRRCM	0.652
	FVAAVSLIV	1.244	HSPSGRSPK	1.093	NIREEMRRL	0.619
	YTVSFTPNI	1.155	EMRRLQRRK	0.795	RRLQRRKQL	0.636
ATAQ	SLFAVLFVV	1.422	SLMATRLLK	1.644	APQRQTSRV	1.302
	ILIPAVIVV	1.201	TTYIVTPVK	1.532	IPAVIVVIL	1.239
	KTTYIVTPV	1.223	VIVVILLIK	1.017	TPVKKASSK	0.839

CTL=Cytotoxic T-lymphocyte

### Prediction of B-cell epitopes

The presentation of antigens triggers antibody production and memory B cell activation through linear B cell epitopes in the adaptive immune system. The ABCPred server was used to predict B-cell epitopes and the 09 highest-scoring B-cell epitopes were selected for use in vaccine design (Table-4).

**Table-4:** Selected B-cell epitopes of three *Rhipicephalus microplus* proteins.

Target protein	Epitope	Score (0.5)
Bm86	CYCPWKSRKPGPNVNI	0.94
	CKCPDDHECSREPAKD	0.91
	TANCSAAPPADSYCSP	0.91
Subolesin	PPTRAHQIDPSPFGDV	0.92
	SEEIAANIREEMRRLQ	0.90
	ICERMMKERESKIREE	0.95
ATAQ	CQARVECNEEEESSCE	0.81
	GQECVYKDGKASCQCP	0.83
	SFCPPGTTGNGSICTN	0.86

### Design of a multi-epitope vaccine

Nine HTL cell epitopes, 18 CTL epitopes, and nine B-cell epitopes with scores >0.51 were chosen for use in the final vaccine design. An appropriate linker was used to connect these epitopes. The 50S ribosomal L7/L12 (P9WHE3) adjuvant was linked with HTL epitopes through the EAAAK linker to enhance the vaccine’s immunogenicity. We used the linkers AAY, KK, GPGPG, and EAAAK to separate each epitope. Consequently, the full multiepitope vaccine design contained 674 amino acid residues in addition to one EAAAK linker, 18 GPGPG linkers, nine AAY linkers, and nine KK linkers. The final construction of the multi-epitope vaccine epitope list is given below (Table-5).

**Table-5:** List of predicted B- and T-cell epitopes.

Target protein	B cells		MHC-I	MCH-II
Bm86	CYCPWKSRKPGPNVNI	A2	YTVSFTPNI	IALFVAAVSLIVECT
	CKCPDDHECSREPAKD		LLNEYYYTV	GIALFVAAVSLIVEC
	TANCSAAPPADSYCSP	A3	TSIGKEVFK	HPIGEWCMMYPKLLI
			ILNCTQDIK	
		B7	CPSGSTVAE	
			CPWKSRKPG	
Subolesin	PPTRAHQIDPSPFGDV	A2	LLNEYYYTV	AGNYTLRCALRSAGA
	SEEIAANIREEMRRLQ		YTVSFTPNI	GNYTLRCALRSAGAE
	ICERMMKERESKIREE	A3	RMMKERESK	IVIGILIPAVIVVIL
			RMMKERESK	
		B7	NIREEMRRL	
			RRLQRRKQL	
ATAQ Protein	CQARVECNEEEESSCE	A2	FFAPALYIV	NLYGKSLMATRLLKC
	GQECVYKDGKASCQCP		RVWATVAIV	GNYTLRCALRSAGAE
	SFCPPGTTGNGSICTN	A3	TTYIVTPVK	QSAMKNLYGKSLMAT
			ITFTQDQVY	
		B7	APQRQTSRV	
			APQRQTSRV	

### Final construction of the multiepitope vaccine

**Figure-1:**
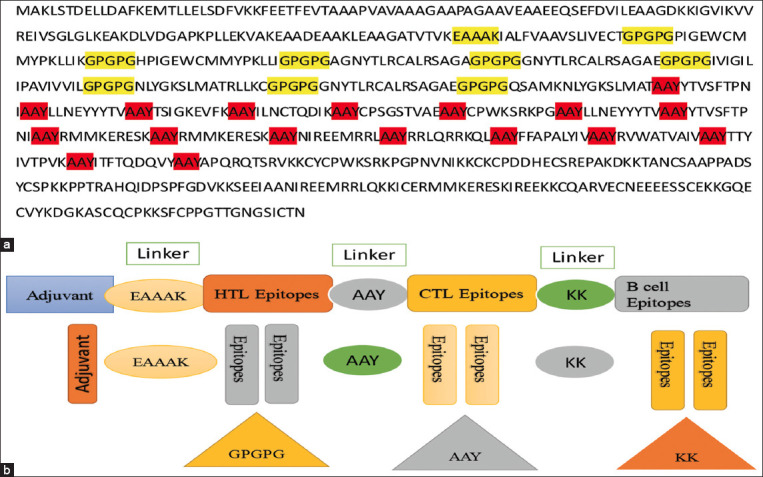
(a) The sequence of a multi-epitope construct with an adjuvant and compatible linkers is given. The EAAAK is the linker for adjuvant, GPGPG is the linker for MHC-II, AAY is the linker for MHC-I, and KK is the linker for B cell epitopes. (b) The design of the construct with compatible linkers.

The final construct of the multi-epitope vaccine was designed and linked with compatible linkers, as shown in Figure-1.

### Physicochemical properties of the proposed vaccine construct

**Figure-2:**
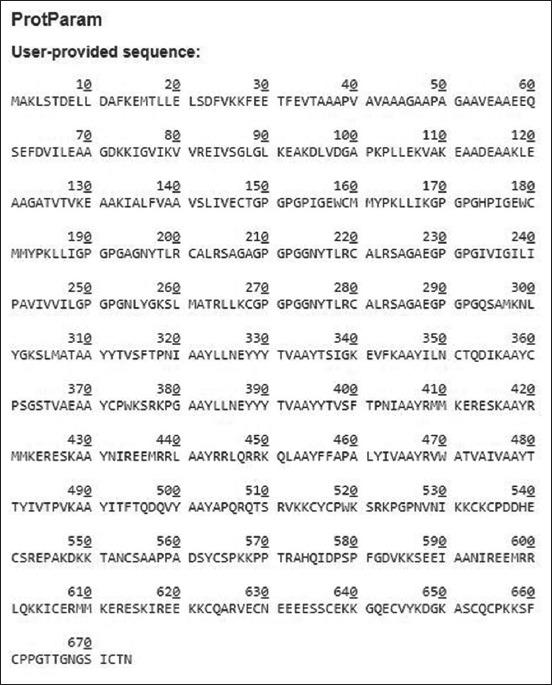
The number of amino acids (674) and physiochemical properties of the designed construct were predicted using the ExPASy Protparam tool. The 674 amino acids of the construct had a 30-h half-life in mammalian cells, >10-h half-life in *Escherichia coli*, and 20 h half-life in *in vitro* yeast. The instability index is <40%, which indicates that the construct is stable and that the hydropathicity GRAVY is −0.227. The construct is hydrophilic.

The physicochemical properties of the final designed construct were evaluated using the ExPASy ProtParam server. The amino acid number, solubility of the construct, half-life, hydropathicity, instability index, and protein structure were evaluated. The 674 amino acids of the construct had a 30-h half-life in mammalian cells, >10-h half-life in *Escherichia coli*, and 20 h half-life in *in vitro* yeast. The instability index is <40%, which indicates that the construct is stable and that the hydropathicity GRAVY is −0.227. The construct is hydrophilic as shown in Figure-2.

### *In silico* cloning

**Figure-3:**
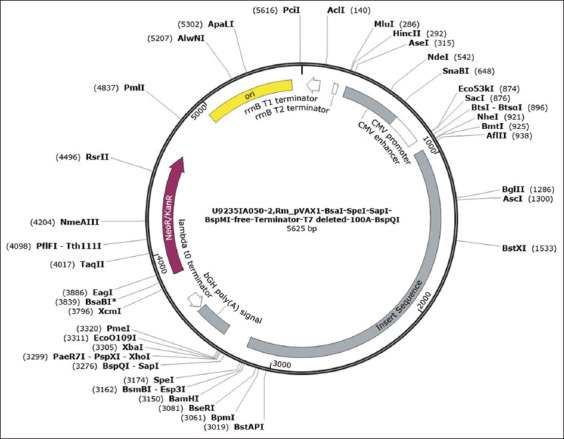
Circular map of designed pVAX1-Rm plasmid. The pVAX1-Rm plasmid has a NeoR/KanR antibiotic-resistant region, and the CMV promotor region and the gene of interest (2227 bp) were cloned between the *BspQ1* and *Nde1* restriction sites.

Using the *in silico* approach, the designed construct was cloned into a compatible vector. The pVAX1-Rm plasmid was designed by cloning the gene of interest; 5′ and 3′ UTRs were added with the gene of interest for stability after *in vitro* transcription. Poly (A) was also added to prevent mRNA degradation after *in vitro* transcription (Figure-3).

### Transformation of the pVAX1 plasmid

**Figure-4:**
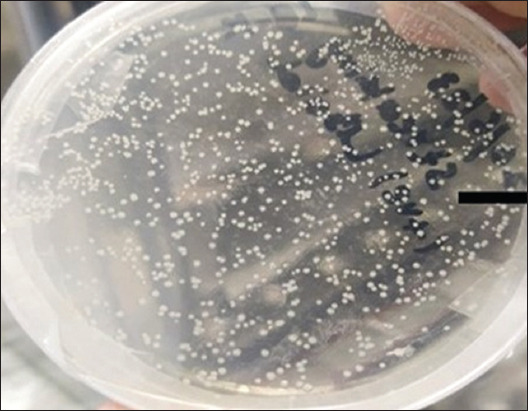
Transformed colonies of pVAX1-Rm plasmid. White colonies indicate the transformed plasmid of pVAX1-Rm in an LB kanamycin (50 μg/μL) agar plate. 200 μL culture was poured on an agar plate and kept overnight in an incubator at 37°C, and transformed white colonies were observed.

The pVAX1 plasmid was transformed in Dh5α competent cells through heat shock (Figure-4).

### Plasmid extraction and digestion

Plasmid extraction was performed using a GeneJET Plasmid Miniprep Kit (Cat. No: 2680476, Thermo Fisher Scientific). The extracted plasmid was run on 1% agarose gels and visualized using Gel-Doc (Ibright750, Thermo Fisher Scientific).

### *In vitro* transcription

The linearized plasmid was transcribed into mRNA using a T7ultra mMESSAGE mMACHINE kit (Cat. No: AM1345, Thermo Fisher Scientific). Enzyme RNA polymerase was used for the *in vitro* transcription of the plasmid into mRNA. The structure of mRNA after *in vitro* transcription is presented below.

### mRNA purity and cDNA synthesis from mRNA

**Figure-5:**
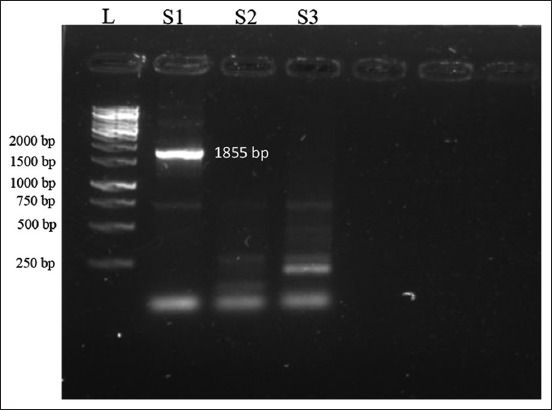
L is a 1-kb DNA ladder (Cat. No: SM0311, GeneRuler, Thermo Fisher Scientific, USA), and S1 is an 1855-bp product of the complementary DNA (cDNA) sample obtained using gene-specific primers. S2 and S3 are samples obtained using oligo primers provided with the kit. Initially, the plasmid was transcribed into messenger RNA and then cDNA was prepared. Then, using gene-specific primers, a 1855-bp final product was obtained.

In the initial step, mRNA was obtained from the transcription of a plasmid and then purified using a MEGAclear purification kit (Cat. No: AM1908, Thermo Fisher Scientific). The final purified mRNA was subsequently concentrated to a concentration of 77.2 ng/μL which was assessed using a Nanodrop spectrophotometer (Thermo Fisher Scientific). Then, cDNA was synthesized using a Superscript reverse transcriptase kit (Cat. No: 18064-022, Thermo Fisher Scientific) from the mRNA transcribed by T7ultra mMESSAGE mMACHINE and then purified by MEGAclear. The mRNA was converted into first-strand DNA, and then, using gene-specific primers, we prepared second-strand DNA. The final product formed was 1855 bp (Figure-5).

### Characterization of LNPs

**Figure-6:**
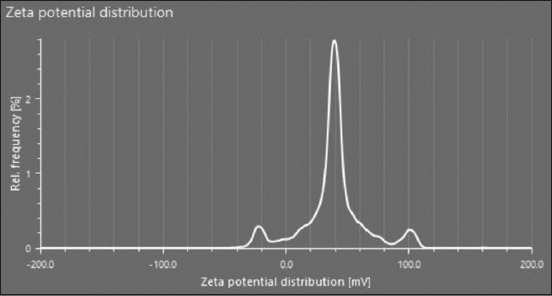
The zeta Potential of lipid nanoparticles with a potential of 37+. The zeta potential predicts the particle charge (37+). Positive-charged lipid nanoparticles can cross the cell membrane and enter the target cell.

It was found that the nanoparticles were morphologically stable and that there was no chemical interaction between the lipid mix and mRNA during the preparation of the LNPs. The prepared particles were nanosized and positively charged (Figure-6). Positively charged cationic ionizable nanoparticles are good carriers of mRNA to target cells.

## Discussion

The number of potential vaccines for combating tick infestation has been determined [15]. A specialized vaccine must be developed to improve the protection and management of tick infestations due to the strain-to-strain diversity of *R. microplus*. One type of specialized vaccine is a multi-epitope vaccine, which has various advantages over other vaccines, such as specificity, stability, and safety, and has been developed against various infectious diseases, including schistosomiasis, toxoplasmosis, and COVID [41]. Several studies have reported on the benefits and experimental validation of multi-epitope vaccinations using immunoinformatic methods [42, 43]. Several vaccines against infectious diseases have been produced using this technique, some of which have since been approved for clinical trials and experimental validation [44–48]. The antigens Bm86 and Subolesin from *R. microplus* can prevent tick infestation when used in vaccine preparation [49–52]. To prepare multi-epitope vaccines, it is important to recognize immunogenic antigens using bioinformatic tools for designing successful protective *R. microplus* vaccines [53]. In this study, the development of a multi-epitope mRNA vaccine against *R. microplus* ticks represents a potential route for the future development of an effective antigenic vaccine. We predicted and used the MHC, HTL, and CTL epitopes of the *R. microplus* proteins that were antigenic, non-toxic, and non-allergic rather than the whole genome. An adjuvant was also used with the construct to increase the vaccine’s immunogenicity. *In silico* cloning of the final construct was performed in a compatible plasmid. After *in silico* cloning, *in vitro* transcription was performed and LNPs of mRNA were prepared and characterized.

**Figure-7:**
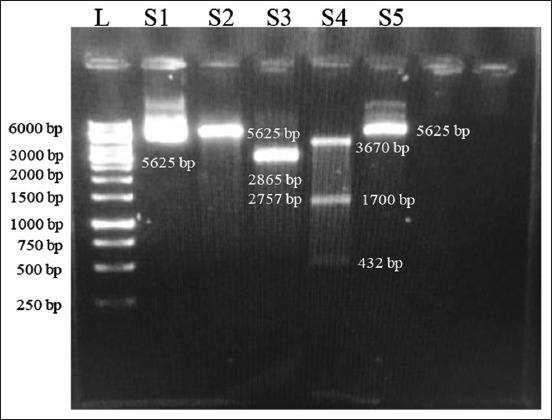
In this figure, the restriction analysis of plasmid was performed, where L is a 1-kb DNA ladder (Cat. No: SM0311, GeneRuler, Thermo Fisher Scientific, USA), S1 is an undigested plasmid (5625 bp), S2 is a linearized plasmid by *BspQ1 (*5625 bp), and S3 is a double-digested plasmid by *Xho1 (*2865 bp) and *Nde1 (*2757 bp). S4 is a digested plasmid by *Acc1* which produces three bands (3670 bp, 1700 bp, and 432 bp), and S5 is an undigested plasmid (5625 bp).

**Figure-8:**
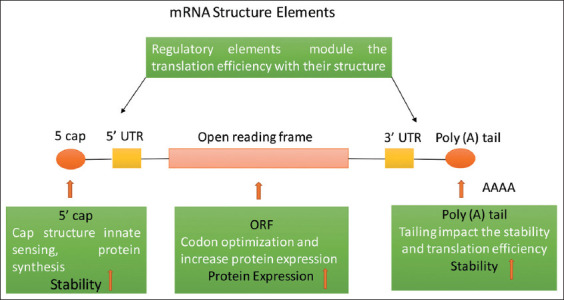
Structure of messenger RNA (mRNA) after *in vitro* transcription. 5’ untranslated region (UTR) and 3’ UTR were added to stabilize the mRNA along with capping and tailing. Capping and tailing avoid extracellular enzyme degradation. The open reading frame region promotes protein expression

Using a variety of immune epitope database tools, HTL, CTL, and B-cell epitopes with an antigenic score >0.5 for target *R. microplus* proteins (Bm86, Subolesin, and ATAQ) were identified and characterized. In a previous study by Younas *et al*. [19], the HTL, CTL, and B-cell epitopes with an antigenic score of >0.5 were also selected. The threshold level for an epitope to be antigenic is 0.5; thus, all selected epitope scores should be >0.5. Overall more than 1000 epitopes were predicted, and from them, only nine HTL epitopes, 18 CTL epitopes, and nine B-cell epitopes of all three proteins were selected with high antigenic scores of 0.958 for Bm86, 0.752 for Subolesin, and 0.964 for ATAQ, respectively, and an adjuvant was used to enhance the immune response (Tables-2–4), all of which were linked to one another using GPGPG, AAY, and KK linkers, respectively, as reported by Naveed *et al*. [54]. Our above results are consistent with those of a similar study in which the epitopes of the target *R. microplus* proteins Bm86, Subolesin, and Bm95 and their antigenic scores were 0.957, 0.752, and 0.946, respectively [19]. The variation in the antigenic score depends on the epitopes used in the construct design. To reduce the likelihood of an autoimmune response occurring, we focused on identifying antigenic and non-allergenic regions within the *R. microplus* strain that were able to interact with various HLA alleles [55]. These antigenic epitopes were connected through linkers, which provide flexibility to the structure of proteins and cause immunogenicity in the designed multi-epitope vaccines. GPGPG, AAY, and KK were included as linkers in previous study by Bazhan *et al*. [56] to prevent the formation of junctional epitopes [56]. In addition, it was found that the EAAAK linker had been included to combine the adjuvant with the remaining epitopes and provide stability, as reported in the previous studies by Narula *et al*. [55] and Shrivastava *et al*. [57]. The physiochemical properties indicate that the instability index of our construct is <40%, which indicates that the construct is stable, and the hydropathicity GRAVY is 0.224, which indicates that our construct is hydrophilic and soluble in water. These results are consistent with a previous study by Hammed-Akanmu *et al*. [19], in which a construct with an instability index below 40% and a GRAVY score of 0.228 was prepared. Negative GRAVY values indicate that the designed construct is stable [18]. The GRAVY score depends on the number of selected amino acids for construct design. After preparation of the final construct, *in silico* cloning was performed in a compatible pVAX1 plasmid for transformation and further *in vitro* transcription. Similarly, *in silico* cloning of the designed construct was performed using compatible plasmids and downstream uses of the plasmid [18]. White colonies of the transformed plasmid were observed (Figure-7). Restriction analysis of the plasmid was performed, and different bands were observed. The *BspQ1* enzymes used to linearize the plasmid and the 5625-bp band were observed. Double digestion of the plasmid by *Xho1* (2865 bp) and *Nde1* (2757 bp) and by *Acc1* which produced three bands of 3670, 1700, and 432 bp (Figure-8). The same results were achieved in which white-transformed colonies and restriction analysis of plasmids containing the enzymes *Xho1* and *Nde1*, as well as 5400 bp and 1456 bp bands were observed [19].

**Figure 9:**
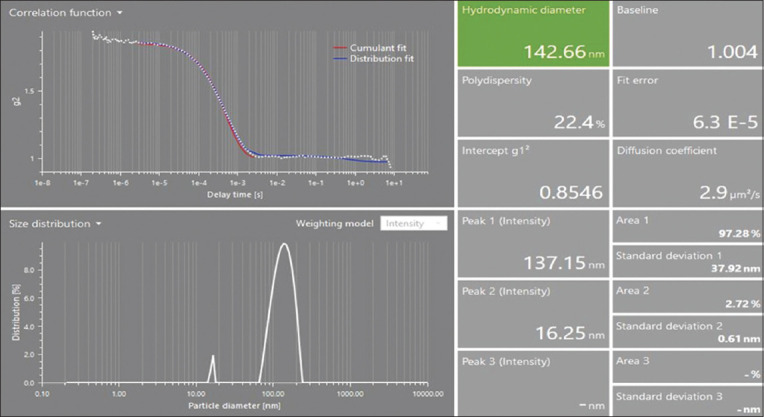
The lipid nanoparticles (LNPs) have an average size of 142.66 nm. The first peak of the LNPs appeared at 137.15 nm, and the second peak intensity appeared at 16.25 nm.

**Figure-10:**
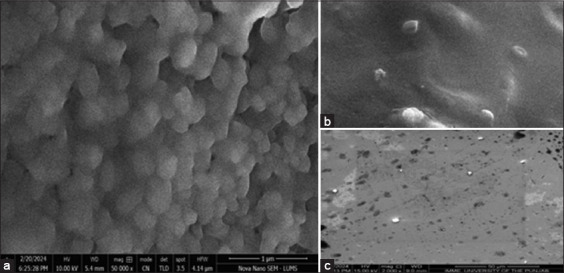
(a) Lipid nanoparticles (LNPs) are in concentrated form at 1 μm and at 50,000× magnification. Their morphology and surface were intact and rod-to-oval shape LNPs, and (b and c) Separated single LNP cells were shown at different magnifications.

The compatible plasmid pVAX1 was used for *in vitro* transcription of the DNA construct into mRNA. The naked mRNA is fragile and can rapidly degrade [58]. To prevent mRNA degradation, LNPs of mRNA were prepared and characterized. It was found that different factors affect the nanoparticle size and charge. LNP size and charge depend on different factors, including the type of lipid, preparation method, concentration, lipid ratio, and LNP mixing method [59]. The 60–200 nm-sized LNPs are promising to deliver to target cells and provoke strong immunity compared with large particle size [59]. In this study, lipid mixtures were developed using four different lipid concentrations (1,2-dioleoyl-3-dimethylammonium-propane50% + DSPC10% + cholesterol 5% + DMG PEG-2000 1.5%), while LNPs were prepared using a ratio of 5:1 of the lipid mixture and mRNA. The average size of our LNPs was 142.66 nm, and the zeta potential was +37 (Figure-9). Higher charges on the particles can predict the stability of the LNPs [58]. Similar results were achieved using lipid mix and mRNA in previous studies by Malburet *et al*. [59] and Hassett *et al*. [60], in which they used 60–800-nm LNPs for immunization and concluded that 60–200-nm particles are excellent for vaccine purposes. The SEM results showed that the shape and morphology of the nanoparticles were intact (Figure-10). However, based on these results, future studies should focus on the efficacy of this multi-epitope mRNA vaccine against *R. microplus* in calves.

## Conclusion

A novel anti-*R. microplus* vaccine was prepared using an immunoinformatics approach. The DNA construct was transcribed into mRNA by *in vitro* transcription, and LNPs were prepared and characterized. The mRNA vaccine-based LNPs will be evaluated for vaccine potential in cattle in the future.

## Authors’ Contributions

MIR: Conceived and designed the study. UU: Designed and analyzed the data and performed the experiments. UU and MIR: Analyzed and interpreted the data and drafted and revised the manuscript. UU, KA, and WS: Data analysis and validation. All authors have read, reviewed, and approved the final manuscript.
